# Putting the “dynamic” back into dynamic functional connectivity

**DOI:** 10.1162/netn_a_00041

**Published:** 2018-06-01

**Authors:** Stewart Heitmann, Michael Breakspear

**Affiliations:** QIMR Berghofer, Brisbane, Australia; QIMR Berghofer, Brisbane, Australia; Metro North Mental Health Service, Brisbane, Australia

**Keywords:** Dynamic functional connectivity, Nonlinear dynamics, Metastability, Multistability

## Abstract

The study of fluctuations in time-resolved functional connectivity is a topic of substantial current interest. As the term “dynamic functional connectivity” implies, such fluctuations are believed to arise from dynamics in the neuronal systems generating these signals. While considerable activity currently attends to methodological and statistical issues regarding dynamic functional connectivity, less attention has been paid toward its candidate causes. Here, we review candidate scenarios for dynamic (functional) connectivity that arise in dynamical systems with two or more subsystems; generalized synchronization, itinerancy (a form of metastability), and multistability. Each of these scenarios arises under different configurations of local dynamics and intersystem coupling: We show how they generate time series data with nonlinear and/or nonstationary multivariate statistics. The key issue is that time series generated by coupled nonlinear systems contain a richer temporal structure than matched multivariate (linear) stochastic processes. In turn, this temporal structure yields many of the phenomena proposed as important to large-scale communication and computation in the brain, such as phase-amplitude coupling, complexity, and flexibility. The code for simulating these dynamics is available in a freeware software platform, the Brain Dynamics Toolbox.

## Author Summary

The study of network fluctuations in time-resolved functional connectivity is a topic of substantial current interest. However, the topic remains hotly disputed, with both positive and negative reports. A number of fundamental issues remain disputed, including statistical benchmarks and putative causes of nonstationarities. Dynamic models of large-scale brain activity can play a key role in this field by proposing the types of instabilities and dynamics that may be present. The purpose of the present paper is to employ simple dynamic models to illustrate the basic processes (“primitives”) that can arise in neuronal ensembles and that might, under the right conditions, cause true nonlinearities and nonstationarities in empirical data.

## INTRODUCTION

The brain is a dynamic machine par excellence, tuned through the principles of self-organization to anticipate the statistics and movement of the external milieu (K. Friston, 2013; Skarda & Freeman, 1987). Its unceasing dynamics and cycle of prediction-action-perception mark it as distinct from even the most advanced deep learning platforms despite impressive advances in machine learning. Systems neuroscience is likewise incorporating dynamic algorithms into its core methodologies (Breakspear, 2017; K. J. Friston, Harrison, & Penny, 2003), in the design of hierarchical models of perception and inference (Mathys, Daunizeau, Friston, & Stephan, 2011); dynamic approaches to clinical disorders (Roberts, Friston, & Breakspear, 2017); dynamic models of functional neuroimaging data (Stephan et al., 2008; Woolrich & Stephan, 2013); and dynamic frameworks for the analysis of resting state fMRI data (Deco, Jirsa, & McIntosh, 2011). Dynamic models are at the heart of the distinction between functional connectivity and effective connectivity (see Box 1; K. J. Friston, 2004) and can help disambiguate correlated activity due to mutual interactions from that caused by input from a common source.

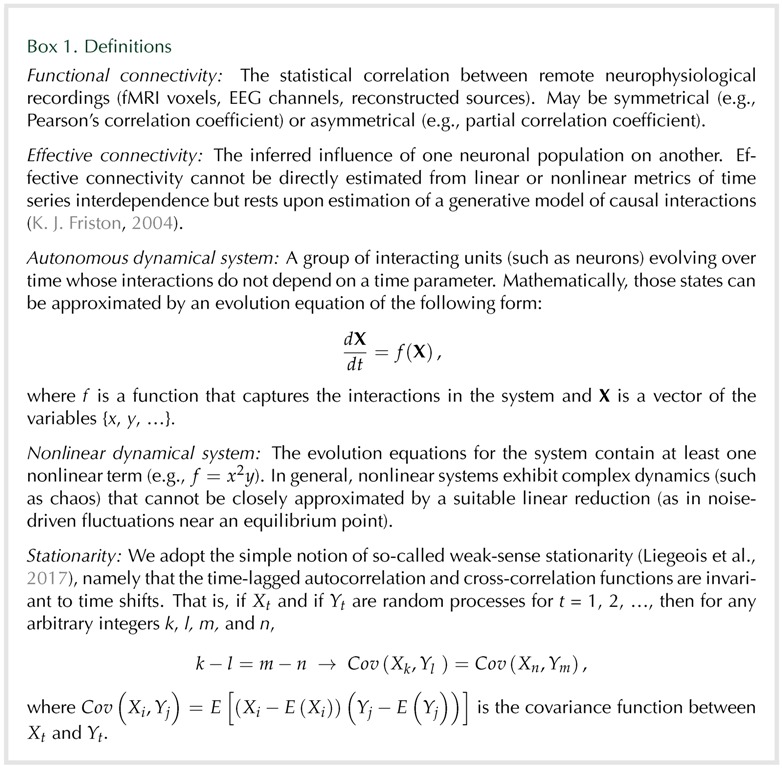

Research into the dynamics of resting-state fMRI data is currently very active, and takes its form largely through the study of nonstationarities in time-resolved functional connectivity (Chang & Glover, 2010; Hutchison et al., 2013; Zalesky, Fornito, Cocchi, Gollo, & Breakspear, 2014). However, the topic remains hotly disputed, with both positive (Abrol et al., 2017; Nomi et al., 2017; Zalesky et al., 2014) and negative (Laumann et al., 2016) reports. In addition, fundamental statistical issues continue to be contested, including the utility of sliding-window analyses (Hindriks et al., 2016; Leonardi & Van De Ville, 2015; Zalesky & Breakspear, 2015) as well as core definitions of stationarity (Liegeois, Laumann, Snyder, Zhou, & Yeo, 2017). Another issue of substance pertains to the *causes* of putative nonstationarities (assuming they exist); in particular, whether nonstationarities reflect subtle cognitive processes (random episodic spontaneous thought, i.e., “rest”; Breakspear, Williams, & Stam, 2004); whether they are slower processes that nonetheless retain cognitive salience (such as drifts in attention and arousal; Kucyi, Hove, Esterman, Hutchison, & Valera, 2016); or whether they are nuisance physiological and head motion covariates that have been inadequately removed from fMRI time series (Laumann et al., 2016). Regardless of these debates, the overarching motivation of the field is that resting-state brain activity is endowed with functionally relevant complex neuronal dynamics—either as the substrate for ongoing “thought,” or to prime the cortex for perception and action (K. Friston, Breakspear, & Deco, 2012). So, the central question seems not whether such neuronal dynamics exist, but to what extent they can be detected in functional neuroimaging data.

Dynamic models of large-scale brain activity can play a key role in this field by proposing the types of instabilities and dynamics that may be present (Cabral, Kringelbach, & Deco, 2014; Deco, Jirsa, McIntosh, Sporns, & Kötter, 2009; Gollo, Zalesky, Hutchison, van den Heuvel, & Breakspear, 2015; Hansen, Battaglia, Spiegler, Deco, & Jirsa, 2015; C. J. Honey, Kötter, Breakspear, & Sporns, 2007). The purpose of the present paper is to employ simple dynamic models to illustrate the basic processes (“primitives”) that can arise in neuronal ensembles and that might, under the right conditions, cause true nonlinearities and nonstationarities in empirical data. In doing so, we also aim to disambiguate some key terms in the field: first, the differences between nonstationarity and nonlinearity—both can herald underlying dynamics, cause rejection of common nonparametric nulls, and (as we will see) occur individually or together; second, the distinctions between key terms in dynamic systems theory, especially the catchphrase terms of metastability and multistability (which are often used interchangeably). Hopefully this is a constructive step toward a more definitive resolution of the uncertainties in the field.

## METHODS

### Coupled Dynamical Systems

To illustrate the breadth of synchronization dynamics, we study an autonomous, nonlinear system of coupled neural masses. This model has been previously employed to study whole-brain dynamics (C. J. Honey et al., 2007; Zalesky et al., 2014). The system is composed of local subsystems (“neural mass,” or nodes) coupled together to form a larger ensemble (for a review, see Breakspear, 2017). Each local node comprises a population of excitatory neurons and a slow variable incorporating the (simplified) response of a local inhibitory pool of neurons. Inhibitory activity is driven by local excitatory activity, to which it feeds back via a slow inhibitory current. The dynamics of neural masses are determined by a conductance-based process, with fast (instantaneous) sodium membrane currents and slower potassium currents. The dynamics within each node takes the form of a low-dimensional nonlinear differential equation,

$$\frac{dX}{dt}=f_{a}\left(X\right),$$ (1)

where **X** is a vector of the system’s variables (cell membrane potentials, firing rates, membrane channel currents). The system has a number of physiologically derived time-invariant parameters *a*, such as synaptic connection strengths, membrane channel conductances, and neural gain (Breakspear, Terry, & Friston, 2003). Depending upon the choice of these parameters, single-node dynamics may range from a steady-state fixed-point attractor, to fast periodic oscillations and chaos. Here we choose the parameters so that the autonomous behavior of each neural mass is described in mathematical terms as a nonlinear dynamical system with a chaotic attractor. This chaotic regime arises from the intrinsic constants and variables within the local (uncoupled) neural population—specifically from the mixing of the fast timescales of the pyramidal cells and the slow responses of the inhibitory population. These dynamics do not depend upon the coupled interactions.

A mesoscopic neural ensemble is constructed by permitting two or more of such local neural masses {**X**_1_, **X**_2_, …} to interact through a coupling function (Breakspear & Stam, 2005). These interactions are parameterized by the matrix of internode coupling **C** = {*c*_*ij*_}, where *i* is the source node and *j* is the receiver node. Connections may be reciprocal but asymmetric (*c*_*ij*_ ≠ *c*_*ji*_). Hence each node’s dynamics are governed by

$$\frac{dX_{i}}{dt}=f_{a}\;\left(X_{i}\right)+H_{c_{ij}}\;\left(X_{j}\right)\;,$$ (2)

where *i* indexes the node and the coupling function *H* embodies the nature of the internode influences among all nodes in the system, that is, the model of effective connectivity. Internode coupling in this framework is classically composed of excitatory-to-excitatory connectivity. However, there are no restrictions on the general nature of Equation 2 that prohibit inhibitory internode coupling parameterized in **C**.

For simulations of empirical macroscopic network behaviors, the connectivity **C** between neural masses can be defined by structural connectivity (Sporns, Tononi, & Kötter, 2005) representing the network of white matter fiber tracts mediating internode connectivity in the brain. The structural connectomes can be obtained from postmortem tracing studies (Stephan et al., 2001) or from in vivo human MRI-based tractography. Because of technical limitations of current state of the art tractography, connectivity matrices derived from MR-based tractography are symmetric *c*_*ij*_ = *c*_*ji*_.

Although we employ a particular model to illustrate synchronization dynamics, many of the underlying principles hold for any local system with chaotic dynamics (Pikovsky, Rosenblum, & Kurths, 2003). Periodic dynamics permit a narrower range of dynamic scenarios. For most of our simulations, we focus on dyads (pairs) of coupled nodes. Complex dynamics on motifs with three or more nodes derive from the principles of two nodes, but add an additional layer of complexity, depending on their connectivity as well as the nature of axonal time delays (Atay, 2010; Cabral, Luckhoo, et al., 2014; Deco et al., 2009; Gollo & Breakspear, 2014; Gollo, Mirasso, Sporns, & Breakspear, 2013). For the moment, we do not consider the role of time delays in the resulting synchronization dynamics. We return to these issues below.

All simulations in this paper are performed using the Brain Dynamics Toolbox (https://bdtoolbox.blogspot.com.au/), an open-source Matlab-based toolbox for interactive simulations of neuronal dynamics (as described in Supplementary Information II, Heitmann & Breakspear, 2018). The Brain Dynamics Toolbox allows scaling up to simulate large ensembles, the employment of other local node dynamics, the introduction of local stochastic influences, and the treatment of internode time delays. Readers may also wish to explore The Virtual Brain (Leon et al., 2013; Sanz-Leon, Knock, Spiegler, & Jirsa, 2015), an open-source Python-based toolbox specifically designed for simulating whole-brain dynamics according to the principles explored here.

### Quantifying and Testing Time Series Dynamics

The detection and quantification of the linear correlations or nonlinear interdependence in time series data rests upon two related steps: (a) the employment of a metric that captures these (linear or nonlinear) properties; and (b) the application of a statistical test to ascertain whether the value of this metric is statistically significant according to an appropriate null hypothesis. The second of these steps recognizes the fact that such metrics are never exactly zero when applied to noisy time series of finite length. In this paper, we use the method of surrogate data to achieve the latter goal. Both steps are now described in further detail.

#### Dynamic metrics.

We employ two metrics of internode interactions: the traditional Pearson’s correlation coefficient, and a measure of nonlinear interdependence based upon time series forecasting methods (Schiff, So, Chang, Burke, & Sauer, 1996; Terry & Breakspear, 2003). These are sensitive to stationary linear correlations (traditional time-averaged functional connectivity) and stationary nonlinear interdependence, respectively. The latter estimates a (normalized) prediction error based upon forward projections of each system’s dynamic trajectory: It approaches 0 for highly structured, completely predictable nonlinear time series and diverges quickly toward a maximum error of 1 when the time series have no structure. Crucially, the measure is sensitive to nonlinearities in the time series, possessing higher values for nonlinear time series than for random time series with the same (cross- and autocorrelation) linear properties. There are two versions: Self-predictions are sensitive to nonlinearities within a time series, whereas cross-predictions are sensitive to nonlinear interdependences between subsystems.

Estimates of dynamic, instantaneous interactions are obtained by examining the behavior of phase differences between time series. The Hilbert transform is first applied to each system’s time series, allowing an estimate of the instantaneous phase (and amplitude) of a signal (Tass et al., 1998). The Hilbert transform of a time series *x*(*t*) is given by

$$Y(t)=\frac{1}{\pi }\int \frac{x(\tau )}{t-\tau }d\tau ,$$ (3)

which can be used to compose the analytic signal,

$$\Lambda (t)=x(t)+iY(t)=A(t)e^{i\varphi (t)},$$ (4)

which uniquely defines the instantaneous amplitude *A*(*t*) and phase *φ*(*t*)of the signal *x*(*t*). Phase dynamics between two signals *x*_*i*_(*t*) and *x*_*j*_(*t*) are then given by

$$\varphi (t)=\left[\varphi_{i}(t)-\varphi_{j}(t)\right]\text{mod}\;2\pi .$$ (5)

#### Surrogate algorithms.

In finite length, autocorrelated time series, measures of (linear and nonlinear) sample correlations are generally not 0, even for uncoupled, independent systems. Measures of correlation taken from large numbers of samples do center at 0, but the variance across individual samples can be substantial. To perform statistical inference on the typically modest number of data available, it is thus necessary to compare empirical measures of coupling to a null distribution derived from ensembles of surrogate data: These are pseudo time series derived from empirical data by resampling methods that preserve the time series length, autocorrelation structure, and amplitude distribution but have had the property of interest (nonstationarity, nonlinearity) destroyed. If the empirical measure falls outside of the null distribution, then the data can be inferred to contain that property of interest.

For the present study, we employ a nonparametric phase-randomization method (Theiler, Eubank, Longtin, Galdrikian, & Doyne Farmer, 1992). Briefly, multivariate data are mapped into the frequency domain by application of the Fourier transform. The phase of each frequency is then independently rotated by a random increment between 0 and 2*π*. The data are then transformed back to the time domain. By leaving the amplitude of each frequency untouched, this process preserves the power spectrum of the time series and hence the linear autocorrelations. By rotating the phases of different time series (in a multivariate stream) by the same random increment, the cross-correlations are also preserved (Prichard & Theiler, 1994). An additional step restores the amplitude distribution of the original time series, which is otherwise rendered Gaussian (Schreiber & Schmitz, 1996). This resampling approach can be adapted for complex three-dimensional data enclosed within a bounded spatial domain, such as whole-brain fMRI, by using the wavelet transform (Breakspear, Brammer, Bullmore, Das, & Williams, 2004).

Phase randomization works because trajectories in smooth continuous dynamical systems (Equation 1) generate time series with highly structured phase relationships across frequencies. To test for significant linear cross-correlations, we simply shift the time series relative to one another (thus preserving auto- but destroying cross-correlations) and test the original against the correlations from the time-shifted surrogate data. To test for nonlinearities within a single time series, we perform phase randomization and compare the nonlinear self-prediction errors of the original time series to the ensuing surrogate distribution. Finally, to establish nonlinear interdependence, we apply a multivariate phase randomization and compare the nonlinear cross-predictions of original and surrogate ensemble.

## RESULTS

We first explore the emergence of dynamic synchrony between two interacting neural masses, each with three dynamical variables **X**(*t*) = {*V*, *W*, *Z*} exhibiting local chaotic dynamics. Specifically, we examine the dynamics of two uncoupled nodes, then two nodes with strong and weak coupling. We plot and analyze the time series corresponding to the average membrane potential of each system, *V*_1_ and *V*_2_. In later sections, we consider the principles underlying larger ensembles and the translation of these dynamics into the setting of noisy experimental data.

### Uncoupled Systems

**Figure 1.**
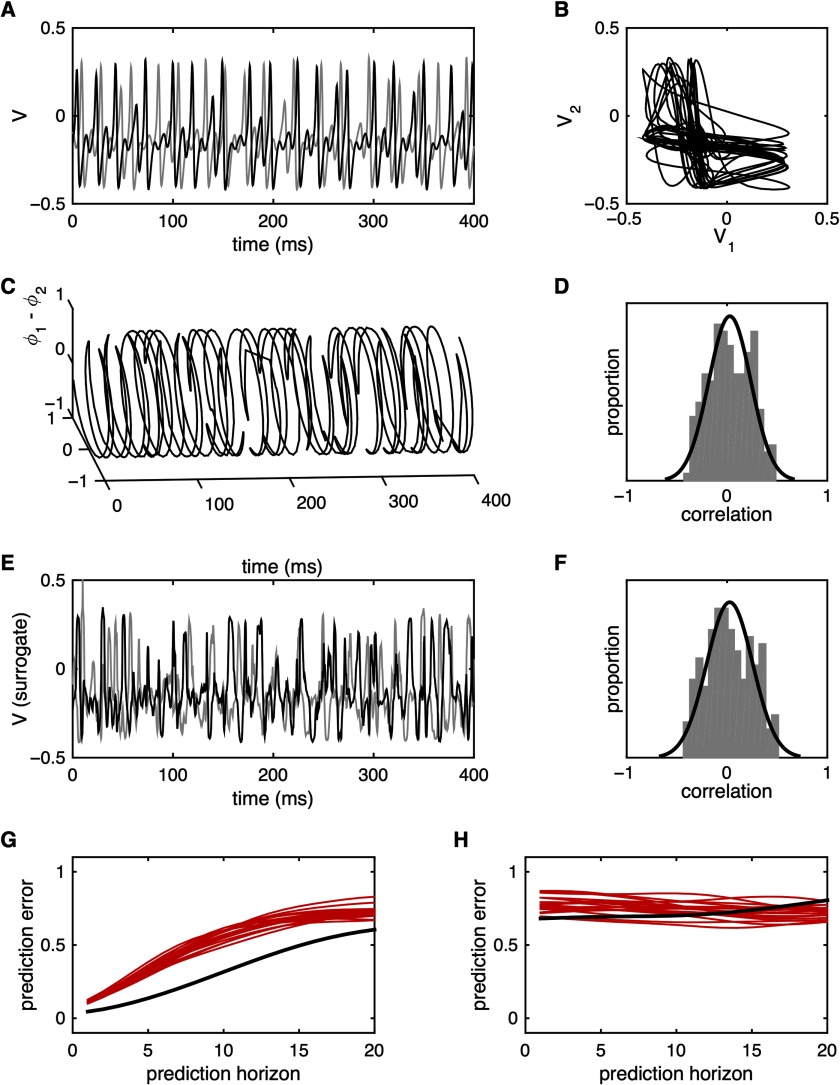
Uncoupled systems. (A) Time series for two uncoupled neural masses (*V*_1_ is black, *V*_2_ is gray) in the chaotic regime. (B) The same time series with *V*_1_ plotted against *V*_2_. Transients (*t* < 100) have been omitted. (C) Hilbert phase of *V*_1_ relative to *V*_2_. Plotted in cylindrical coordinates with unit radius. (D) Distribution of linear correlations between *V*_1_ and *V*_2_ for multiple simulation runs with random initial conditions. (E) Amplitude-adjusted surrogates for the time series from panel A. (F) Distribution of linear correlations between surrogate data drawn from the same instances of *V*_1_ and *V*_2_ (i.e., one simulation run, multiple shuffles of the surrogate data). (G) Nonlinear self-prediction of V1 from itself (black) and from surrogate data (red). Note that both errors grow toward one with longer prediction horizons, but the original data falls well below the null distribution. (H) Nonlinear cross-prediction of *V*_1_ from *V*_2_ (black) and from surrogate data (red). Here the empirical data falls within the surrogate distribution, reflecting the absence of intersystem coupling.

In the absence of coupling *c*_*ij*_ = *c*_*ji*_ = 0, the two coupled neural subsystems evolve independently (Figure 1). Because of their intrinsic aperiodic dynamics, the two systems evolve in and out of phase even if their parameters are identical. Plotting the time series of one system *V*_1_ directly against the other *V*_2_ reveals the lack of any underlying synchronization structure (Figure 1B). As a result, the difference between the two systems’ phase (modulus 2*p*) unwinds (Figure 1C). It is, however, important to note that because of the autocorrelations within each time series, the linear correlation coefficient is often not close to 0 for any particular finite length sample: The correlation coefficient for the time series shown in Figure 1A is 0.08. However, the distribution of the linear correlation coefficient from an ensemble of repeated realizations of the time series is centered at 0 (Figure 1D). This is a reminder that anecdotal observations of nonzero correlations can easily be misinterpreted as functional connectivity in the data, where there is none.

Surrogate data generated from the time series in Figure 1A by (multivariate) phase randomization are shown in Figure 1E. The distribution of linear correlations between time series generated by repeated application of phase randomization are shown in Figure 1F: It can be seen that the empirical correlation (0.08) falls within the surrogate distribution. This observation confirms that the ensemble of surrogate data does adequately represent the null distribution of trivial linear correlations that arise because of the finite sample length.

Do these data contain further (i.e., nonlinear) structure? This can be tested by studying the nonlinear prediction errors, specifically how forward projections of one system’s orbits predict the actual evolution of either that same system (nonlinear self-prediction error) or the other system (nonlinear cross-prediction error; Schiff, So, Chang, Burke, & Sauer et al., 1996; Terry & Breakspear, 2003). Because this approach is based upon a low-dimensional phase space reconstruction, it is sensitive to nonlinear, as well as linear, correlations within the data. Here we see that such forward predictions (of one system predicting itself, Figure 1G, and of one system predicting the other, Figure 1H) are less than their theoretical maximal value of 1 (black lines). The nonlinear (self-) prediction errors fall well below the forward predictions arising from surrogate data (red lines), because the original time series have internal nonlinear structure, arising from the local chaotic dynamics. However, the nonlinear cross-prediction errors fall within the null distribution, because there is no coupling and thus no nonlinear interdependence.

In sum, uncoupled chaotic neuronal dynamics give rise to autocorrelated time series with trivial linear cross-correlations that distribute around 0. Nonlinear self-prediction errors lie outside the null distribution, confirming that each time series contains nonlinear (chaotic) structure. However, nonlinear cross-prediction errors fall within the null distribution generated by surrogate data that contain the same linear correlations. That is, these data arise from independent (uncoupled) stationary nonlinear processes.

### Generalized Synchronization

**Figure 2.**
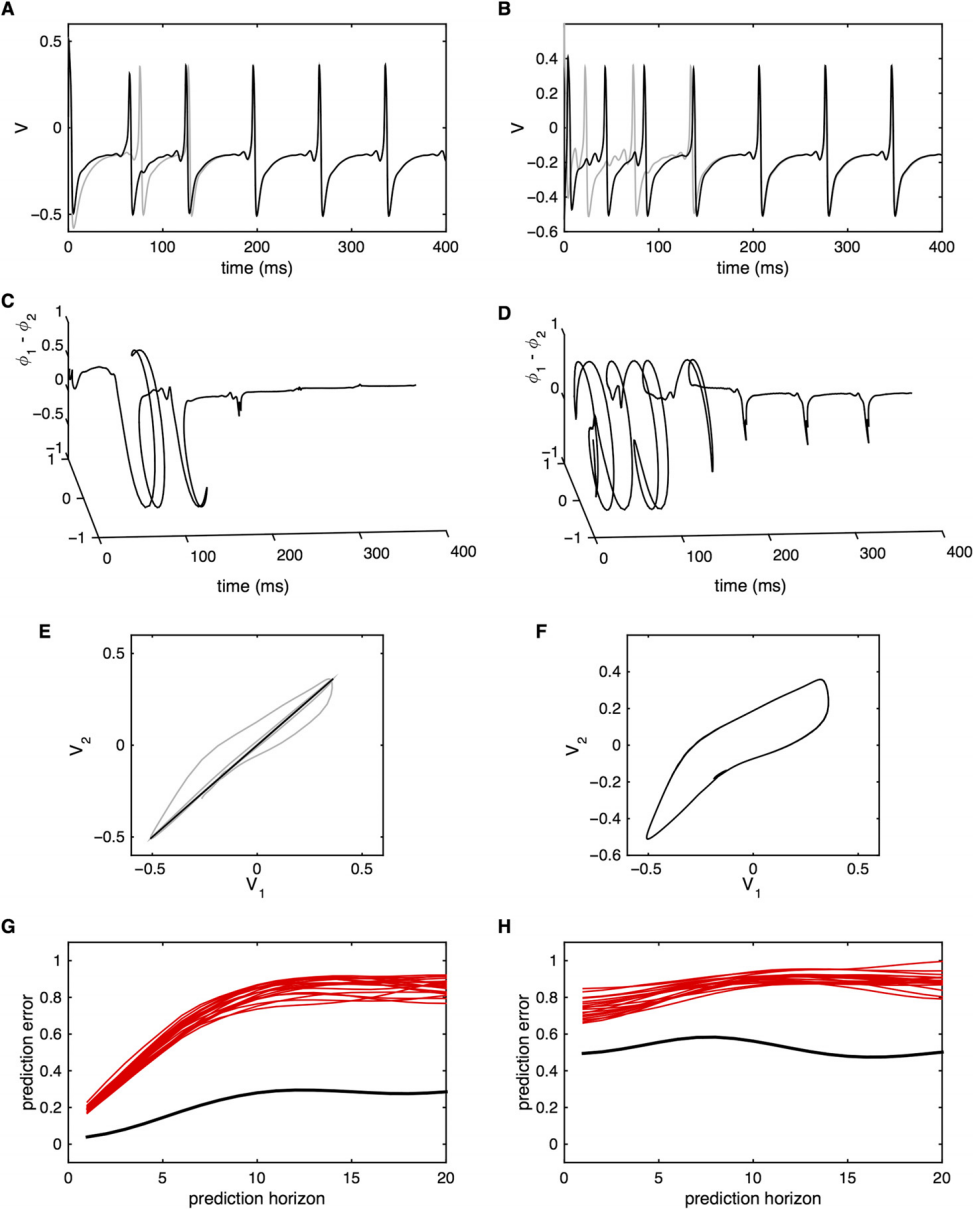
Generalized synchrony. (A) Time series for two coupled identical neural masses (*V*_1_ is black, *V*_2_ is gray) exhibiting identical synchronization. (B) Time series for two coupled nonidentical neural masses (*V*_1_ is black, *V*_2_ is gray) exhibiting generalized synchronization. (C) Hilbert phase of *V*_1_ relative to *V*_2_ for the case of identical synchronization. Note the rapid approach to stable 1:1 phase synchrony. (D) Hilbert phase of *V*_1_ relative to *V*_2_ for the case of generalized synchronization. Brief, but incomplete, phase slips continue to occur following the transient. (E) *V*_1_ plotted against *V*_2_ for the cases of identical synchronization. After a brief transient, the system approaches the diagonal. (F) *V*_1_ plotted against *V*_2_ for the cases of generalized synchronization. Transients have been omitted. (G) Nonlinear self-prediction of *V*_1_ from itself (black) and from surrogate data (red). (H) Nonlinear cross-prediction of *V*_1_ from *V*_2_ (black) and from surrogate data (red).

In the presence of strong unidirectional coupling, such as *c*_12_ = 0.6, *c*_21_ = 0, two neural subsystems with identical parameters exhibit a rapid convergence to complete synchrony; that is, the second (slave) system rapidly adjusts its dynamics to match those of the first (master) system (Figure 2). Thereafter the two systems pursue identical orbits; that is, they exhibit identical synchronization, evidenced by their rapid convergence to perfect phase synchrony (Figure 2C), and their states approach the hyperdiagonal in phase space, *V*_1_ = *V*_2_, *W*_1_ = *W*_2_, *Z*_1_ = *Z*_2_. For simplicity, we plot a two-dimensional cross section through the full dimensional phase space spanned by *V*_1_ and *V*_2_ (Figure 2C). It can be seen that the initial transient (gray line) rapidly converges onto the hyperdiagonal (black line).

The onset of identical synchrony occurs for much weaker internode coupling if it is bidirectional, *c*_12_ = 0.05, *c*_21_ = 0.05. This is because both systems are able to simultaneously adjust their internal dynamics according to the state of the other system, leading to a more stable, integrated system.

Biological systems are obviously not composed of identical subsystems because some degree of asymmetry is inevitable. However, two neural masses with modestly mismatching parameters continue to exhibit strong, rapid, and stable synchrony if the internode coupling is sufficiently strong, for example, *c*_12_ = 0.6, *c*_21_ = 0 (Figure 2B). These dynamics are accompanied by stable 1:1 phase locking between the two systems (Figure 2D). That is, following an initial transient of phase unwinding (until *t* = ∼150 ms), the phase difference remains close to 0, although it shows brief, bounded excursions. Rather than contracting onto the (hyper-) diagonal linear subspace, the orbits of this system converge toward a smooth manifold that lies just off the diagonal (Figure 2F). This phenomenon, known as generalized synchronization, arises in a broad variety of coupled asymmetric chaotic systems (Afraimovich, Verichev, & Rabinovich, 1986; Hunt, Ott, & Yorke, 1997; Pecora & Carroll, 1990; Rulkov, Sushchik, Tsimring, & Abarbanel, 1995). The smooth surface onto which the orbits converge is known as the synchronization manifold.

The time series generated in this scenario embody several instructive properties. The presence of synchrony gives rise to linear correlations that are close to unity. After a brief transient of less than 150 ms, the correlation coefficient is above 0.99 for all successive time windows. That is, the system has stationary linear cross-correlations. In the presence of static measurement noise, such a system would give rise to stationary functional connectivity (that is, the ensemble linear statistics are stationary over successive time windows). However, these time series also contain deeper structure than multivariate surrogate data that possess the same linear (auto- and cross-) correlations. That is, the nonlinear prediction error (Figure 2G) and nonlinear cross-prediction (Figure 2H) of the original data are both smaller than prediction errors of the corresponding linear null distributions. This arises because the system traverses phase space on the highly structured and smooth synchronization manifold.

In addition to the presence of stationary linear statistics, these data thus contain nonlinear correlations previously termed “dynamic connectivity” (Breakspear, 2004). This property of the data permits rejection of the null hypothesis represented by the multivariate surrogate data, namely that the time series are generated by a stationary multivariate linear process. Since trivial analysis of the stable and very high linear correlations shows that the linear statistics are stationary, then the preceding analyses point to the (true) alternative hypothesis that the data are generated by a stationary multivariate nonlinear process.

### Metastability

**Figure 3.**
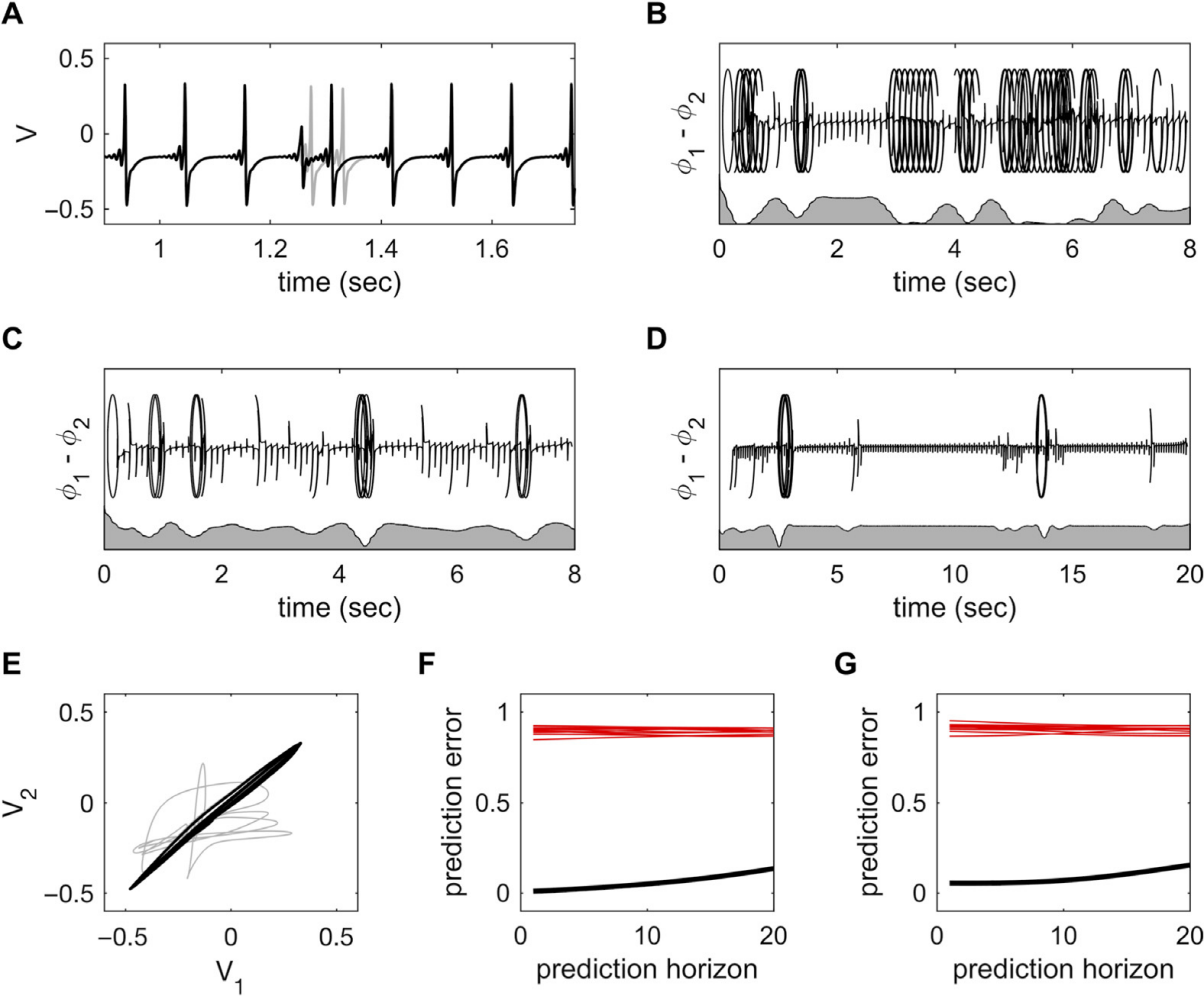
Metastability. (A) Time series for two weakly coupled neural masses (*V*_1_ is black, *V*_2_ is gray) showing a single instance of desynchronization. (B) Hilbert phase of *V*_1_ relative to *V*_2_ with relatively weak coupling. Periods of generalized synchronization are interspersed by erratic desynchronization. The gray shaded region (bottom of panel) shows the point-wise correlations between *V*_1_(*t*) and *V*_2_(*t*) smoothed over a 1-s moving window. (C) Hilbert phase of *V*_1_ relative to *V*_2_ with medium coupling. The instances of desynchronization have become relatively infrequent and briefer. (D) With strong coupling, instances of desynchronization are relatively rare. (E) Plot of *V*_1_ versus *V*_2_ for the case of strong coupling. The desynchronization is seen as a brief, erratic excursion from the synchronization manifold. (F) Nonlinear self-predictions of *V*_1_ from itself (black), and (G) nonlinear cross-predictions of *V*_1_ from *V*_2_ (black). Predictions of *V*_1_ from surrogate versions of *V*_2_ are shown in red. The time series retain nonlinear structure despite the instances of desynchronization.

We next study the development of generalized synchrony in the presence of increasingly strong unidirectional coupling *c*_12_ > 0, *c*_21_ = 0, that is, as the second system gradually adjusts its dynamics to those of the first. Increasing coupling *c*_12_ from 0 leads to a monotonic increase in the time-averaged correlation coefficient until the onset of stable generalized synchronization. However, the accompanying dynamic behavior is quite complex (Ashwin, Buescu, & Stewart, 1996). When the coupling is not sufficiently strong, the two systems show instances of desynchronization, evident as a separation of the states of each system (see example in Figure 3A) and a complete unwinding of the relative phase. For weak levels of unidirectional coupling (e.g., *c*_12_ = 0.1), brief periods of generalized synchrony (and corresponding phase locking) appear among longer intervals of phase unwinding (Figure 3B). If the coupling is increased, the duration of synchronous epochs lengthens, and the instances of phase unwinding become confined to brief, erratic bursts (Figure 3C). Even in the presence of reasonably strong coupling (e.g., *c*_12_ = 0.5),such bursts continue to (infrequently) appear if one waits for a sufficiently long period of time (e.g., a single burst over a 20-s duration, Figure 3D). Meanwhile, as the coupling increases, the synchronization manifold contracts toward the hyperdiagonal, with asynchronous bursts corresponding to brief, disorganized, large amplitude excursions (Figure 3E).

The occurrence of such bursts corresponds to a dynamical phenomenon known as metastability. In brief, for strong coupling, the system possesses a single, low-dimensional chaotic attractor that is embedded within the synchronization manifold: Although the dynamics of this chaotic attractor are reasonably complex (Supplementary Information I, Heitmann & Breakspear, 2018), both systems converge onto the same manifold, corresponding to stable (and stationary) generalized synchronization (Ashwin, 1995). The dynamics considered within the full (six-dimensional) space spanned by both systems become relatively simple. However, if the coupling is slowly weakened from this scenario, there appears a critical value *c*_*k*_ below which instabilities appear within the synchronization manifold and the system “blows out” into the full phase space for brief instances (this is formally called a blowout bifurcation; Ashwin, Aston, & Nicol, 1998; Ott & Sommerer, 1994). In the vicinity of this blowout bifurcation *c*_12_ ≈ *c*_*k*_, the intervals between asynchronous bursts can be very long, following a heavy-tailed process (Ott & Sommerer, 1994).

Metastability is perhaps better known when there are multiple competing states (Cocchi, Gollo, Zalesky, & Breakspear, 2017; M. Rabinovich, Huerta, & Laurent, 2008; M. I. Rabinovich, Huerta, Varona, & Afraimovich, 2008). Such a system cycles between such states, exhibiting a broad variety of synchronous behaviors (such as a variety of cluster solutions; Ashwin & Field, 1999). In the present setting, there is only one such unstable state and the system hence jumps away, then returns back toward the same synchronous state. This specific type of behavior is known in the literature as itinerancy (Kaneko, 1985; Kaneko & Tsuda, 2003; Tsuda, 2001). In more technical parlance, it is an example of homoclinic itinerancy (“homo” referring to a single system that is both attracting and repelling).

Itinerancy endows the time series with highly nonstationary properties: The unstable bursts yield a loss of phase synchrony and a corresponding local decrease in the linear correlation coefficient, both of which return to high values during the longer periods of generalized synchronization. As a result, fluctuations in time-dependent linear correlations from the original time series are greater than those arising from multivariate (stationary) surrogate data. Nonlinear prediction errors and cross-prediction errors both remain outside the null distributions (from multivariate surrogate data) even if these are obtained from long windows that contain several of the bursts (Figures 3F and 3G).

A final summary description of these data is therefore quite nuanced. Recall that they are generated by a coupled nonlinear dynamic system whose parameters are all constant and, in particular, do not depend upon time. These data are hence generated by an autonomous, multivariate nonlinear process. They yield data whose nonlinear properties (for example, phase locking) are highly dynamic. The linear properties of these dynamics are also highly nonstationary; that is, they possess fluctuating time-resolved functional connectivity. Moreover, because the itineracy has long-tailed (non-Poisson) statistics, these properties cannot be captured by a classic finite state Markov model and hence may, in certain circumstances, violate formal definitions of weak-sense stationarity (Liegeois et al., 2017).

The term “dynamic functional connectivity” is arguably a poor term to summarize these properties and to disambiguate metastability from the stationary but nonlinear properties that arise in the setting of generalized synchronization, both of which permit rejection of the stationary, linear null. We return to this issue in the Discussion section.

### Multistability

We consider one further dynamical scenario that yields nontrivial, dynamical interdependence between two or more systems, namely multistability. In a multistable system there exist two or more stable attractors. That is, there are dynamical regimes that, in the absence of noise, trap the behavior of a system indefinitely. Spontaneous switching between the coexisting states then arises when there is noise *ζ* added dynamically to the states,

$$\frac{dX_{i}}{dt}=f_{a}\left(X_{i}\right)+H_{c_{ij}}\left(X_{j}\right)+b.\zeta_{i},$$ (6)

where *ζ*_*i*_ is a stationary zero mean stochastic process scaled in amplitude by the parameter *b*. When the noise is of sufficient amplitude, a multistable system is able to escape the basin of each attractor, and jump from one to the other. This is a subtle, albeit important, difference between multistability and metastability. A metastable system is composed of only unstable nodes, and the evolution of the system cycles from one to the other (or back to itself) even if there is no noise *ζ* = 0. In contrast, a multistable system will settle onto one stable attractor unless external noise is injected *ζ* > 0. The difference may seem subtle but the mechanisms, emergent system behavior, and resulting statistics are quite distinct (for a review, see Cocchi et al., 2017).

In an array of coupled systems such as we are considering, multistability can arise when each individual node has multiple attractors. It can also emerge when the individual nodes are monostable, but the internode interactions introduce multiple types of synchronization dynamics (Ashwin & Field, 1999). In the system considered above, there is only one (chaotic) attractor per node but the coupled ensemble can exhibit multistable attractors, for example when there are three or more nodes and their interactions have axonal time delays (Gollo & Breakspear, 2014).

The emergence of multistability through the interactions of monostable elements is very interesting, but also rather complex. For reasons of relative simplicity, we will thus illustrate a system of coupled nodes where each single node has two attractors; a fixed point and a co-occurring periodic limit cycle. That is, each individual node can exhibit either steady state or oscillatory behavior, depending on the state to which it is initially closest. A simple—or “canonical”—form of this system has been used to model the human alpha system (Freyer, Roberts, Ritter, & Breakspear, 2012) and is a mathematical approximation to a complex neural field model (Freyer et al., 2011). The equation for the amplitude dynamics of a single node according to this simplified model are given by

$$\frac{d\text{r}}{dt}=-r^{5}+\lambda r^{3}+\beta r+b_{1}.\zeta_{1}+b_{2}.\zeta_{2}r,$$ (7)

where *r* is the amplitude, *λ* and *β* are parameters that control the size and depth of the fixed-point and limit-cycle attractor basins. The parameters *b*_1_ and *b*_2_ control the influence of the additive *ζ*_1_and multiplicative noise *ζ*_2_*x*, respectively (see Supplementary Information III for full details; Heitmann & Breakspear, 2018).

**Figure 4.**
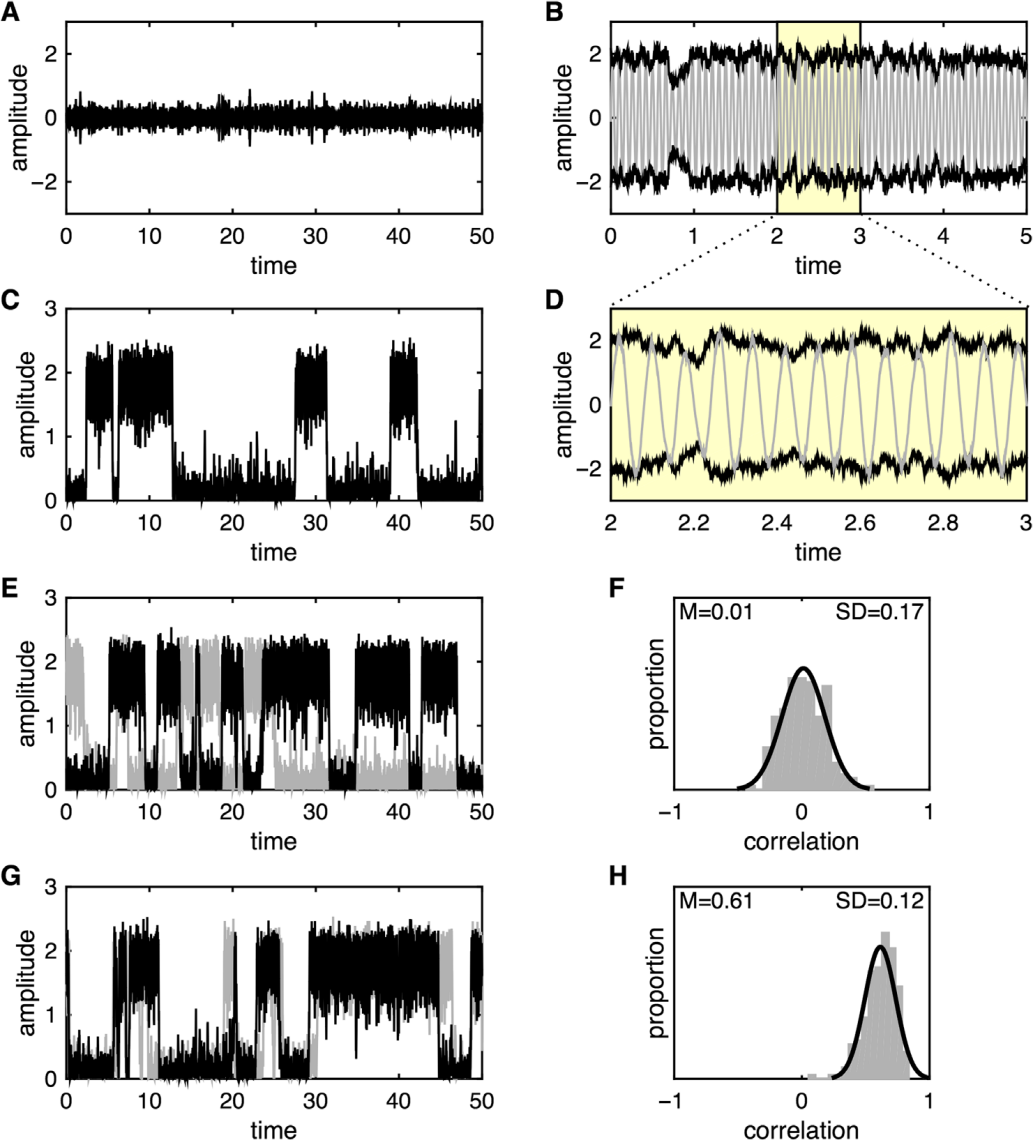
Multistability. (A) Time series of the noisy subcritical Hopf model with one node. With *β* = −10 the system exhibits a stable (noise perturbed) fixed point at *r* = 0. (B) With *β* = −6 the system exhibits a stable limit cycle with amplitude *r* = 2. Oscillations are shown in gray. Black represents the noise-driven amplitude fluctuations, with close-up shown in panel D. (C) With *β* = −7.5, the system exhibits bistability with noise-driven switching between the fixed point and limit cycle. For simplicity, the (gray) oscillations are not shown. (E) System with two nodes and *β* = −7.5 but zero coupling (*c* = 0). The systems jump between the fixed point and limit cycles independently. (F) Histogram of the linear correlations between the time series generated by the two nodes from panel E. The simulation was repeated for *N* = 200 trials with random initial conditions for each trial. The correlations center at 0 but with substantial intertrial variability. (G) System with two nodes and *β* = −7.5 and strong coupling (*c* = 1). The jumps between the fixed point and limit cycles occur in similar time windows. (F) Histogram of the linear correlations between the time series generated by the two nodes from panel E. The correlations center well above 0 with reduced intertrial variability.

When the attractor basins of each system are large (i.e., the basin boundaries are distant from the attractors) and the noise has low amplitude, the two coupled systems exhibit either noise-driven low-amplitude fluctuations (Figure 4) or high-amplitude oscillations (Figure 4B). When the noise is of sufficient strength or the attractor basins are shallow, the dynamics at each node jump from one attractor to the other. In the absence of internode coupling, these transitions occur independently (Figure 4C). The introduction of intersystem coupling increases the coincidence in the timing of the state transitions (Figure 4E). However, because of the presence of system noise, these do not always co-occur, even for relatively strong coupling.

To illustrate the corresponding interactions between two coupled multistable nodes, we focus on their amplitude fluctuations and ignore their phases. In the absence of coupling (*c*_*ij*_ = *c*_*ji*_ = 0), linear correlations between the amplitude fluctuations converge toward 0 for sufficiently long samples. However, linear correlations taken from brief samples do fluctuate considerably. Locally, the noise-driven amplitude fluctuations are highly incoherent because the noisy inputs are independent (i.e., *ζ*_*i*_ ≠ *ζ*_*j*_). However, if the two systems do transition, by chance at similar times, then the local linear correlations are driven by these large amplitude changes in the variance, giving rise to large (but spurious) correlations (both positive and negative). Over time, these fluctuations center on 0 (Figure 4F), although they have high variance (*SD* = 0.17) as a consequence of coincidental state switches. Moreover, the distribution of sample correlations (taken from short time windows) is not substantially influenced if one of the time series is randomly shifted in time compared with the other: The distribution of values is thus a reflection of the stochastic timing of the erratic amplitude jumps within each system, and whether both systems happen to switch within the same time window.

In the presence of coupling, the local fluctuations remain uncorrelated. This is due to the independence of the noise sources, *ζ*_*i*_ ≠ *ζ*_*j*_. Even though the function *f* is nonlinear, the system evolves in a largely linear fashion within each attractor, and the intersystem coupling is overwhelmed by the independent noisy perturbations around each attractor. However, if one system jumps between basins, it then exerts a strong pull on the other system, until it too jumps to the corresponding attractor. The ensuing coincidence of such large-amplitude state changes then skews the sample linear correlation toward the right (i.e., positively) so that they center at a value greater than 0 (Figure 4H). Linear correlations from long time series converge to a positive value that is typically larger than the average of the sample correlations, because such long windows are increasingly dominated by the large-amplitude state changes. Notably, the average of the sample correlations and the long-term correlation coefficient converge toward 0 if one time series is independently rotated in time with respect to the other, underscoring the effect of intersystem coupling on sample correlations.

As raised above, the local (very short-term) fluctuations are dominated by the independent noise sources, even in the presence of coupling. These data do not contain additional nonlinear structure (both the nonlinear prediction errors and cross-prediction errors fall within the null). Between state transitions, the data resemble stationary stochastic fluctuations. Only when considered on lengthy time series data do the sample statistics reflect the presence of the underlying nonlinear multistable attractor landscape.

The time series generated by a coupled (noise-driven) multistable system hence show multivariate statistics that are *locally* stochastic, independent, and stable, but are *globally* highly correlated and fluctuate substantially. If the noise term is independent of the state of the system (as per Equation 6), then the switching between attractors is Poisson (Freyer et al., 2012). The statistics of the time series can then be closely approximated by a finite state Markov process, with a fixed likelihood Λ of jumping states at any time, thus generating Poisson statistics with an exponential distribution of dwell times. Despite the erratic nature of the state transitions, this result theoretically renders the statistics weak-sense stationary (WSS) because the expected correlation and cross-correlations are independent of time (Liegeois et al., 2017).

However, there is one final nuance that is conceptually important. In many situations, the influence of the state noise *ζ* is state dependent, in which case a more general differential equation pertains:

$$\frac{dX_{i}}{dt}=f_{a}\left(X_{i}\right)+H_{c_{ij}}\left(X\right)+G_{b}\left({X,\zeta }_{i}\right),$$ (8)

where the influence of the state noise *ζ* is dependent on the states **X** via the function *G*. When the noise is state dependent, (e.g., *G*_*b*_(**X**, *ζ*_*i*_) = *b***X**.*ζ*_*i*_, as in the case of Figure 4), then the system typically gets *trapped* near each of the attractors in a nonstationary manner (Freyer et al., 2012). More technically, in a setting of purely additive noise, transitions probabilities are time invariant and follow a stationary Poisson process. But with multiplicative noise, the chance of a state transition decreases as the time since the last transition increases. This nonstationarity gives rise to a heavy-tailed (stretched exponential) distribution of dwell times (Freyer et al., 2012). Long dwell times are more likely than in the case of purely additive noise. More crucially, the dwell time is dependent on the history of the system. As a consequence, sample statistics cannot be well approximated by a standard finite state Markov process. This is a system for which the covariance between the two nodes is not time invariant and the process is thus *not* weak-sense stationary.

In sum, the system governed by Equation 6 for *c*_*ij*_ > 0 yields stochastic (linear) time series that fluctuate considerably. However, the statistics are only nonstationary in the strict sense if the noise is multiplicative (state dependent) so that system gets trapped within each state and the ensuing statistics are non-Poisson, which better resemble the statistics of physiological fluctuations.

### Complex Dynamics in Larger Ensembles

We have thus far restricted our analyses to coupled dyads in order to illustrate dynamic “primitives”—generalized synchronization, metastability, and multistability. However, cognitive function inevitably involves exchanges between a substantial number of cortical regions—certainly more than two (Frässle et al., 2017; Razi & Friston, 2016; Seghier & Friston, 2013; Sporns, 2010). To what extent do dynamics in dyads inform our understanding of dynamics in larger ensembles, particularly as time delays (*τ*) between nodes become an indispensable part of modeling larger systems?

In some circumstances, the complex dynamics that occur between two nodes are inherited “upwards” when a large array of nodes are coupled together using the same principles of coupling. Thus, a system expressing multistability during the interaction between two nodes will often exhibit noise-driven multistable switching when more nodes are added. In this situation, nodes may cluster into “up” and “down” states; that is, nodes may cluster into similar states within the same attractor basin, likewise segregated from other clusters which co-occupy a distinct state. In fact, in many coupled oscillator systems, such multistable clustering is quite generic (Hansel, Mato, & Meunier, 1993) and can theoretically encode complex perceptual information (Ashwin & Borresen, 2005).

On the other hand, introducing more nodes can lead to additional complexities and dynamic patterns that are not possible with two nodes. A classic example is the nature of phase relationships between nodes in the presence of time-delayed coupling: With two nodes, the time delays cause a phase lag between the coupled nodes’ oscillations. However, when three nodes are coupled in an open chain (or “V”) formation, then the outer nodes can exhibit stable zero-lag synchrony, with the middle node jumping erratically between leading and lagging the outer nodes (Vicente, Gollo, Mirasso, Fischer, & Pipa, 2008). Although first described in arrays of coupled lasers (Fischer et al., 2006), considerable work has since shown that such zero-lag configurations arise in small V-shaped motifs of coupled neural systems, including spiking neurons (Vicente et al., 2008) and neural mass models (Gollo, Mirasso, Sporns, & Breakspear, 2014). Importantly, stable zero-lag synchrony between the outer nodes of a V motif can survive immersion into larger arrays, where they increase the stability of the system as a whole (Gollo et al., 2015). Such observations support the notion that these coupled triplets underlie the emergence of zero-lag correlations that have been observed in diverse neurophysiological recordings (Gray, König, Engel, & Singer, 1989; Singer & Gray, 1995). However, closing the three-node motif by adding a link between the outer nodes (hence turning the V into a cycle) destroys stable zero-lag synchrony, instead promoting “frustrated” metastable dynamics (Gollo & Breakspear, 2014).

Time delays can generate many complex phenomena at the network level—especially when the time delays are heterogeneous—even when the uncoupled (individual) nodes have linear or limit-cycle behaviors (Atay, 2010). In addition to the emergence of metastability (Hansel et al., 1993), time delays can also introduce slow collective frequencies (i.e., ensemble oscillations that are much slower than the frequencies of the uncoupled individual units; Cabral, Kringelbach, et al., 2014, Niebur, Schuster, & Kammen, 1991). Other complex dynamics that can emerge through the influence of time delays include traveling waves (Breakspear, Heitmann, & Daffertshofer, 2010) and chimera states—ensemble dynamics whereby there is a domain of coherent nodes and a separate domain of chaotic, incoherent nodes (Abrams & Strogatz, 2004; Breakspear, Heitmann, & Daffertshofer, 2010; Laing, 2009; Shanahan, 2010).

While considerable progress has been made in this area, the full armory of complex dynamics in large systems of coupled neural subsystems is far from understood. For illustrative purposes, we consider a number of candidate scenarios that arise in larger arrays, specifically when simulating neural mass dynamics on a matrix of 47 cortical regions derived from CoCoMac (Stephan et al., 2001), a compilation of tracing studies from the macaque brain that yields a sparse (22%) binary directed graph (available in the Brain Connectivity Toolbox, Rubinov & Sporns, 2010). We employ a coupling function $H_{c_{ij}}$ that mimics a competitive (agonist) feedback between local self-excitation and input from distant regions such that as the influence of external nodes is scaled up by a coupling constant *c*, local recurrent feedback is correspondingly scaled down by (1 − *k*). All internode influences occur through the coupling matrix *C*_*ij*_.

**Figure 5.**
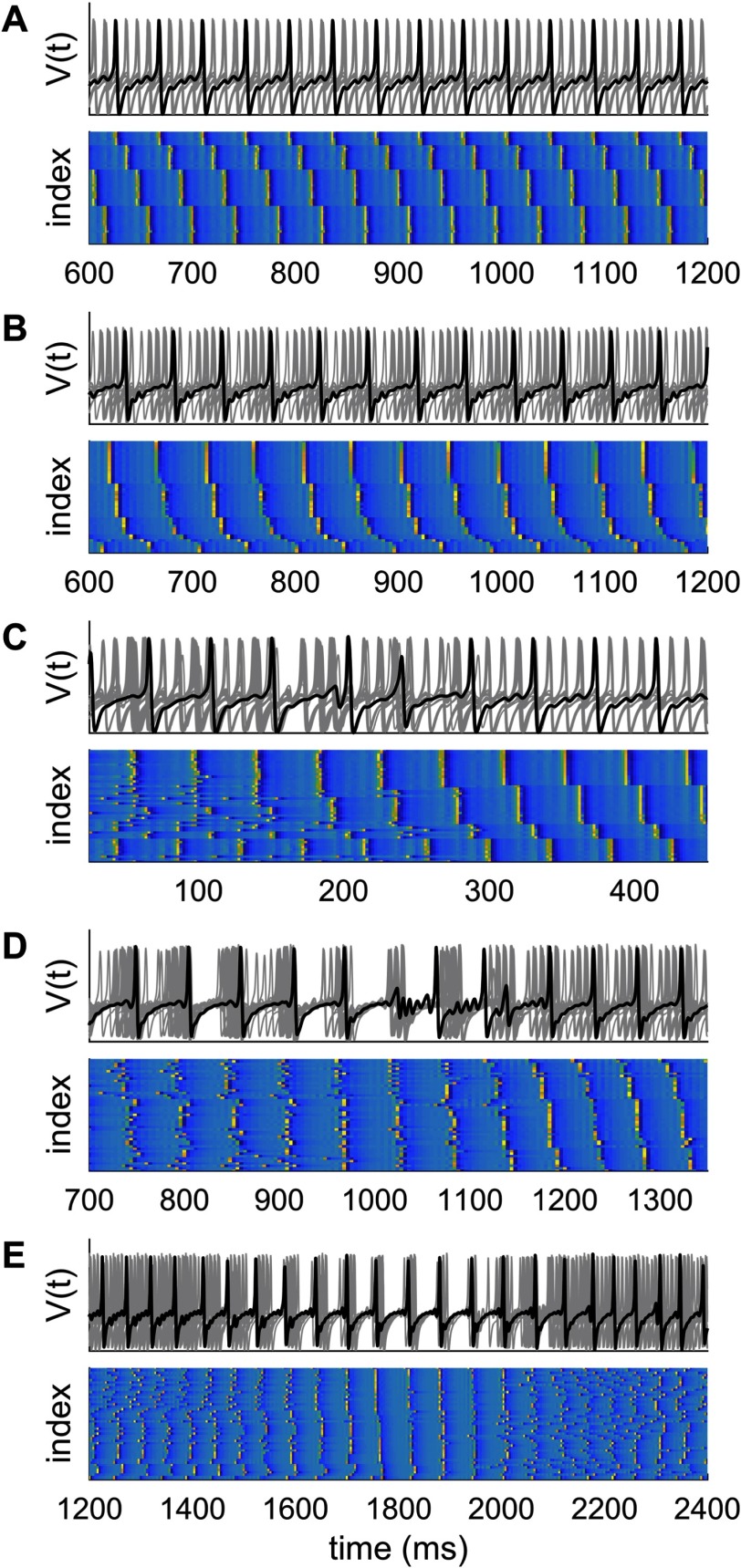
Complex dynamics in larger ensembles. (A) Stable partitioning of ensemble dynamics into four phase-coupled clusters with *τ* = 10 ms and coupling *c* = 0.75. (B) Partitioning of ensemble dynamics into six phase-coupled clusters with *τ* = 6 ms and coupling *c* = 0.75. There is slightly greater disorder in some of the clusters compared with those in panel A. (C, D) With weaker coupling and/or shorter time delays (*τ* = 5.5 ms, *c* = 0.45), there are brief phase slips, leading to a reorganization of the cluster configuration. (E) With briefer time delays (*τ* = 5 ms), clustering does not occur. Instead the system shows instances of global synchrony interspersed among spatiotemporal chaos.

The neural mass model employed here possesses two timescales—a fast local field oscillation of approximately 100 Hz nested within a slower timescale of approximately 10 Hz (due to the slow inhibitory feedback; see Supplementary Information I, Heitmann & Breakspear, 2018). When parameterized with strong internode coupling (e.g., *c* = 0.75) and a time delay that approaches the period of the fast oscillations of the neural mass model (*τ* = 6–10 ms), the ensemble dynamics break into a number of phase-coupled clusters (Figure 5).

Each cluster is constituted by phase entrainment to a common beat of the faster rhythm. The full array of cluster states then recur over the course of the slow oscillation. Note that the number of clusters may differ according to the time delay (four clusters for *τ* = 10 ms, Figure 5A; and six clusters are apparent for *τ* = 6 ms, Figure 5B). In this scenario, the nodes within clusters show stable, stationary generalized synchronization. Nodes in different clusters also show generalized synchronization, albeit with a constant phase offset. This is an ensemble equivalent of stable generalized synchrony in coupled dyads.

This example illustrates the self-organization of coupled neural systems into dynamic communities, an example of functional segregation. Of interest, if the coupling is weaker (e.g., *c* = 0.45) or the time delay shorter (*τ* ∼ 5–6 ms), the ensemble dynamics show brief instances of desynchronization, such that the global coherence of regions into clusters decreases and nodes switch alliances between clusters (Figure 5C). Similar occasions of desynchronization can herald a reconfiguration from a poorly organized state to highly clustered dynamics (Figure 5D). In these settings, the ensemble shows some dynamic flexibility in addition to segregation. Such instances render the ensemble statistics nonstationary around the point of transition.

If a shorter time delay (*τ* ∼ 5 ms) is incorporated into the model, then a clean demarcation into distinct phase-coupled clusters does not arise. The ensemble rather shows instances of near global synchrony interspersed by longer periods of relatively dispersed dynamics (Figure 5E). During the epochs of high global synchrony, zero-lag synchrony emerges, and as a result, the ensemble has highly ordered (low entropy) dynamics: Outside of these windows, the amount of order among the nodes is low. The ensemble statistics in this setting are both nonlinear and nonstationary.

If the time delays approach 0, the state of global synchronization becomes increasingly stable, even for very weak couplings—resembling the scenario for coupled dyads (Figure 2). Conversely, if the time delays are increased, the synchronized cluster solutions become unstable. So, there is a finite range of delays (related to the fast dynamics of the neural masses) for which clustering and metastable dynamics occur. In simple systems, such as coupled Kuramoto (phase) oscillators, this relationship can be derived analytically (Yeung & Strogatz, 1999). In systems with nontrivial local dynamics and highly asymmetric connectivity matrices, such as the CoCoMac graph employed here, there are added layers of complexity that remain poorly understood.

These scenarios illustrate the capacity of neuronal ensembles to exhibit a diversity of dynamic behaviors that yield nonlinear and nonstationary statistics. In some scenarios, dynamical primitives that characterize coupled pairs (generalized synchronization, metastability, and multistability) dominate the ensemble dynamics, yielding their characteristic dynamic fingerprints. New phenomena also appear, including zero-lag synchrony (despite the presence of time delays) and clustering. Typically, these new behaviors compliment the basic dynamics present in coupled dyads, hence metastable bursts yielding spontaneous reconfiguration of cluster states.

## DISCUSSION

The growth of interest in “dynamic” resting-state functional connectivity motivates a deeper understanding of synchronization dynamics in neural systems. Our objective was to illustrate the breadth of synchronization dynamics that emerge in pairs and ensembles of coupled neural populations, and to propose these as putative causes of empirical observations. To recap, coupled dyads exhibit several basic forms—dynamic “primitives”—that yield nontrivial statistics in the ensuing time series. Generalized synchronization yields stationary nonlinear time series. Metastable dynamics, which arise when the intersystem coupling is below a critical threshold, yield nonstationary and nonlinear statistics. Multistability yields a nonstationary process that is locally linear (i.e., on short timescales) but evidences strong nonlinear properties globally (over long timescales). When such pairs are integrated into a larger ensemble and the coupling is imbued with time delays, then these basic primitives combine with new phenomena, such as phase clustering, to cause complex dynamics that spontaneously switch between different network configurations. This yields time series whose statistics violate the assumptions of a stationary stochastic process and which hence yield nontrivial fluctuations in time-resolved functional connectivity. The dynamics primitives of generalized synchronization, metastability, or multistability may thus account for the spontaneous fluctuations observed in resting-state fMRI data.

It is also interesting to consider the computational potential of these synchronization dynamics. Neural mass models describe the local average of neural activity, namely local field potentials and average spike rates, not individual spikes. It has been proposed that coherent oscillations in the local field potentials of disparate brain regions promotes information transfer (Palmigiano, Geisel, Wolf, & Battaglia, 2017) and spike time–dependent plasticity (Fries, 2005). Accordingly, the dynamics illustrated in this paper would allow such neuronal binding (Engel, Fries, König, Brecht, & Singer, 1999) to occur across multiple timescales, and among dynamic cortical assemblies that form and dissolve through active, nonlinear processes (Breakspear, Williams, et al., 2004; Kirst, Timme, & Battaglia, 2016). Dynamic synchronization, and desynchronization, could also underlie the spontaneous shifts in attention that co-occur with changes in neuronal oscillations (Jensen, Kaiser, & Lachaux, 2007; Womelsdorf & Fries, 2007) and in the absence of changes in task context (Kucyi et al., 2016). The nesting of a fast oscillation in a slower one—as occurs for our neural mass model—yields phase-amplitude and phase-phase interactions, which have been proposed supporting cognitive processes requiring complex spatial and temporal coordination (Canolty et al., 2006; Miller et al., 2010; Mormann et al., 2005; Tort, Komorowski, Manns, Kopell, & Eichenbaum, 2009). More deeply, the presence of weak instabilities (such as brief desynchronizations) in cortical dynamics has been proposed as a means by which cortex can be primed to respond sensitively to sensory perturbations and thus to optimize perceptual performance (Cocchi et al., 2017; K. Friston et al., 2012). Future work is required to elucidate the effects of exogenous stimulation and endogenous parameter modulation on neural dynamics, and thus to more directly address these issues (Deco & Kringelbach, 2017).

There are several important caveats of the present study. Most notably, functional connectivity denotes correlations between neurophysiological recordings (see Box 1): We have interrogated the time series of simulated neuronal states *X*. Neural states are not directly evident in empirical functional imaging data, which rather arise from noisy and indirect observation processes. We can somewhat crudely represent this as

$$Y_{v}\left(t\right)=M\left(X_{I}\left(t_{T}\right)\right)+\eta_{v},$$ (9)

where *Y*_*v*_ is the empirical signal in channel/voxel *v*, *M* is a complex measurement process (a nonlinear convolution over a set of regions *i* ∈ *I* and time *τ* ∈ *T*), and *η*_*v*_ is the added measurement noise. Functional connectivity is defined as *Cov*(*Y*_*v*_, *Y*_*w*_) not *Cov*(*X*_*i*_, *X*_*j*_). In the case of fMRI, the BOLD signal arises from the summed effect of neuronal activity signaling slow changes in blood flow, mediated by neurovascular coupling (Buxton, Wong, & Frank, 1998). The net effect of this hemodynamic response function (HRF) is a broad low-pass filter (K. J. Friston, Mechelli, Turner, & Price, 2000), which also includes some spatiotemporal mixing if sampled at sufficiently high spatial resolution (Aquino, Schira, Robinson, Drysdale, & Breakspear, 2012). Empirical functional connectivity in rs-fMRI experiments thus reflect slow changes (<0.1 Hz) in synchronization dynamics plus the effect of local spatial mixing. Although we do not explicitly model the observation function, it is worth noting that both meta- and multistability yield fluctuations that are substantially slower than the timescales of the single-node neural dynamics (see Figures 4B–4D), clearly extending into the slow timescales of the HRF. Explicitly incorporating an observation function into a computational framework is crucial to any definitive resolution of fMRI fluctuations. Recent work, using neural mass models that capture the essential internal connectivity and timescales of cortical microcircuits, driving an observational HRF, mark an important step in this direction (Bastos et al., 2012; K. J. Friston et al., 2017). However, the appearance of slow fluctuations in synchrony from fast dynamics lies at the core of the body of work using neural mass models to study fMRI (Cabral, Hugues, Sporns, & Deco, 2011; Deco et al., 2009; Deco & Jirsa, 2012; Gollo, Zalesky, et al., 2015; C. J. Honey et al., 2007; Zalesky et al., 2014).

In comparison, EEG and MEG directly detect field fluctuations and do not suffer the same temporal filtering as fMRI. Analyses of resting-state EEG (Breakspear, 2002; Breakspear & Terry, 2002; C. Stam, Pijn, Suffczynski, & Da Silva, 1999) and MEG (C. J. Stam, Breakspear, van Cappellen van Walsum, & van Dijk, 2003) data using nonlinear time series techniques have shown that the human alpha rhythm is imbued with substantial nonlinear structure. Integrating these findings with use of biophysical models has shown that the alpha rhythm arises from multistable switching between a fixed point and periodic attractor in the presence of multiplicative noise (Freyer et al., 2011; Freyer et al., 2012)—precisely the scenario illustrated in Figure 3. This process yields fluctuations in alpha power whose timescales clearly extend into those of the HRF (Freyer, Aquino, Robinson, Ritter, & Breakspear, 2009). Crucially, the presence of multiplicative noise in the model (and non-Poisson dwell times for each attractor) imply, as discussed above, that the system statistics are history dependent and are not (weak-sense) stationary according to formal, quite restrictive definitions (Liegeois et al., 2017). By this we can infer that the statistics of resting-state cortex *are* nonstationary.

Despite their superior temporal fidelity, EEG and MEG sensor data involve substantial spatial summation of source activity. Although “off the shelf” (Oostenveld, Fries, Maris, & Schoffelen, 2011; Tadel, Baillet, Mosher, Pantazis, & Leahy, 2011) source reconstruction methods are now available, they inevitably incorporate assumptions about the covariance structure of the sources and the measurement noise (Baillet, Mosher, & Leahy, 2001). As such, and despite post hoc unmixing steps (orthogonalization; Hipp, Hawellek, Corbetta, Siegel, & Engel, 2012), there does not yet exist a definitive account of the contribution of synchronization dynamics to source-level M/EEG activity. Given recent accounts of complex multinetwork switching in such data (Baker et al., 2014), substantial steps toward this end seem within reach.

In addition to spatial and temporal filtering, empirical data are also corrupted by extraneous “noise,” including physiological effects (EMG, respiratory confounds) and measurement noise (thermal fluctuations, etc.). These external (nuisance) noise sources (*η* in Equation 9) are conceptually distinct from intrinsic system noise (*ζ* in Equation 7), which are an essential part of neuronal dynamics. Prior assumptions regarding the amplitude and temporal correlation structure of measurement noise are a crucial prelude to a formal Bayes-based model inversion scheme (Stephan et al., 2008). While resolving the contribution of measurement noise to resting-state fluctuations is largely a methodological and empirical issue (Laumann et al., 2016; Uddin, 2017), computational modeling can also assist. As we have seen above, multistable and metastable processes yield specific heavy-tailed statistics. Most nuisance confounds either have a specific timescale (such as respiratory effects; Chang & Glover, 2009), or have very short-range correlations (such as thermal effects). The hope here is to use the statistical fingerprints of synchronization dynamics to help disambiguate true from spurious fluctuations in observed data. Given the salience of brain-body interactions (as indexed by physiological and behavioral interdependences; Allen et al., 2016), it should also be considered that *some* physiological correlates will index true neuronal dynamics (Nguyen, Breakspear, Hu, & Guo, 2016) and not simply artifacts. Computational models that incorporate somatic and physiological dynamics—which thus embody and not merely eschew these signals—may be required here (Petzschner, Weber, Gard, & Stephan, 2017).

Complex network dynamics are a topic of substantial interest (Boccaletti, Latora, Moreno, Chavez, & Hwang, 2006), particularly in computational and network neuroscience (Ashourvan, Gu, Mattar, Vettel, & Bassett, 2017; Khambhati, Sizemore, Betzel, & Bassett, 2017; Sizemore & Bassett, 2017; Stitt et al., 2017). Network fluctuations co-occur with a variety of fluctuating cognitive processes within and across resting-state fMRI sessions (Shine, Koyejo, & Poldrack, 2016; Shine & Poldrack, 2017). To understand how basic dynamics between pairs of coupled systems scale up, we simulated network dynamics on a structural connectome using connectivity data from CoCoMac. This approach—of dissecting network activity into basic synchronization dynamics among coupled dyads (Gollo & Breakspear, 2014; Gollo et al., 2015)—contrasts with the engagement of emergent network dynamics without recourse to the dynamics among the basic elements (Cabral et al., 2011; Deco et al., 2009; Deco & Jirsa, 2012; C. J. Honey et al., 2007; Zalesky et al., 2014). Our simulations showed how dynamical primitives mix with new ensemble phenomena to inform global dynamics, including the presence of clustering and global synchronization. We did not explore the specific role of the CoCoMac connectome network topology in shaping these dynamics, nor correlations between functional and structural connectivity, which has been the subject of substantial prior work (C. Honey et al., 2009). However, modeling work in this field—mirroring empirical resting-state research—has focused on structural correlates of time-averaged functional connectivity. Future work is required to build upon the early forays in this direction by examining multiple timescales in more detail (Cabral, Kringelbach, & Deco, 2017; Deco & Jirsa, 2012; Gollo et al., 2015).

Although it has intuitive appeal, the term “dynamic functional connectivity” is arguably a clumsy one as it suggests, perhaps naively, that dynamic processes exist within the stream of observable data. Dynamics occur in the underlying neuronal system. If they extend into the temporal and spatial aperture of a particular functional neuroimaging modality (in sufficient strength to survive corruption by measurement effects), then they cause nontrivial statistics in time-resolved data samples. From this perspective, it would be preferable to use simple descriptive terms to capture the fluctuating properties of these time-resolved data and reserve the notion of dynamics to refer to their underlying causes.

## AUTHOR CONTRIBUTIONS

Stewart Heitmann: Conceptualization; Formal analysis; Methodology; Resources; Software; Visualization; Writing – original draft; Writing – review & editing. Michael Breakspear: Conceptualization; Formal analysis; Methodology; Supervision; Visualization; Writing – original draft; Writing – review & editing.

## FUNDING INFORMATION

This manuscript was supported by the National Health and Medical Research Council (118153, 10371296, 1095227) and the Australian Research Council (CE140100007).

## TECHNICAL TERMS

Time series: Time-dependent measurements of a dynamic system, such as EEG and fMRI data from the cortex.
Metastability: The behavior of a system without any stable attractors, but rather a sequence of weakly attracting saddles; sets (such as fixed points or periodic attractors) that are attracting in some directions, but are repelling in at least one direction.
Multistability: The behavior of a system with multiple attractors which is then driven by sufficient noise to make the states jump erratically from one attractor to the next. In the absence of noise, the system will settle indefinitely onto one attractor.
Neural mass: A local population of excitatory (and/or inhibitory) neurons whose states are not treated individually, but rather averaged together and treated as a single collective entity.
Chaos: A form of dynamical behavior that can arise from an autonomous nonlinear system. Chaos is characterized by sustained aperiodic (nonrepeating) oscillations, leading to extreme sensitivity of future states to slight changes in present values of the system.
Structural connectivity: Neuronal connections, typically mediated by direct axonal projections from one neuron (or neural population) to another. Structural connectivity can be described at the microscopic (neuronal and neural circuit) through to the large scale macroscopic scale of whole-brain tractography.
Generalized synchronization: Two or more coupled systems exhibit generalized synchronization when there exists a smooth and continuous mapping between the states of one system and the states of the other.
Synchronization manifold: A smooth (continuous and differentiable) surface that lies within a complex system’s phase space, onto which its orbits settle once generalized synchronization is achieved.
