# Supplementary material for: Putting the “dynamic” back into dynamic functional connectivity
Source: Netw Neurosci. 2018 Jun 1;2(2):150–74. doi: 10.1162/netn_a_00041 (PMC6130444; doi:10.1162/netn_a_00041)
Supplement: Supplementary file 1 [file netn-02-150-s001.pdf]

Heitmann, S., & Breakspear, M. (2018). Supporting Information for "Putting the "dynamic" back into dynamic functional connectivity." *Network Neuroscience*, *2*(2), 150-174. https://doi.org/10.1162/netn_a_00041

Supplementary Information I: Neural mass model

Comment [KW1]: COMP: Supplementary info; move to a separate file; not copyedited

Full details of this model can be found in (Breakspear et al., 2003). The version here is adapted from (Roberts et al., 2017).

This simple neural mass model has three state variables: mean membrane potential of local pyramidal cells V, mean membrane potential of inhibitory interneurons Z, and the average number of open potassium ion channels W. Conceptually, as a conductance-based neural mass model, its formulation is similar to the Morris-Lecar single neuron above, but where the quantities are now population means, and with the addition of feedback from a passively enslaved local inhibitory population. The governing equations are,

$$\frac{dV}{dt} = -[g_{Ca} + r_{NMDA} a_{ee} Q_V(V)] m_{Ca}(V)(V - V_{Ca}) - [g_{Na} m_{Na}(V) + a_{ee} Q_V(V)](V - V_{Na}) - g_K W(V - V_K) - g_L(V - V_L) + a_{ie} Z Q_Z(Z) + a_{ne} I_V, \quad \text{(S1)}$$

$$\frac{dZ}{dt} = b[a_{ni} I_Z + a_{ei} V Q_V(V)], \quad \text{(S2)}$$

$$\frac{dW}{dt} = \phi[m_K(V) - W]. \quad \text{(S3)}$$

These are in nondimensional units where capacitance C=1; time is thus also nondimensional but usually considered to be numerically equivalent to milliseconds (Breakspear et al., 2003). Here I_V and I_Z are nonspecific inputs to excitatory and inhibitory populations, respectively; in the absence of noise we set $I_V = I_Z = I_0$. The model distinguishes between AMPA and NMDA channels, where r_{NMDA} denotes the ratio of NMDA receptors to AMPA receptors, and a_{xy} terms parameterize the strength synaptic coupling from population x ($= e, i, n$, where n is a non-specific input) to population y ($= e, i$) (note that this index ordering is the reverse of the physics convention). Parameters b and ϕ are rate parameters (inverse time constants) that determine the time scales of Z and W, respectively. Setting b=0.1 means that the time scale of the inhibitory cells, Z are slow with respects to the pyramidal cells, V.

As in the Morris-Lecar neuron, for the pyramidal neurons the g_{ion} terms are conductances of the corresponding ion channels, and the $m_{ion}(V)$ functions describe the voltage-dependent gating of these ion channels, except that in this mean-field case they describe population-level fractions of open channels. They take the sigmoidal form,

$$m_{ion}(V) = 0.5\left[1 + \tanh\left(\frac{V - T_{ion}}{\delta_{ion}}\right)\right], \qquad \text{(S4)}$$

where T_{ion} is the threshold membrane potential for a given ion channel, and δ_{ion} is the corresponding standard deviation in this threshold. The self-feedback of the

pyramidal cells is split into a conventional voltage-dependent term for sodium channels $a_{ee}Q_V(V)(V - V_{Na})$, and a state-dependent NMDA term, $r_{NMDA}a_{ee}Q_V(V)m_{Ca}(V)(V - V_{Ca})$. This more complex term incorporates the state-dependent nature of NMDA-gated calcium channels.

The voltage-dependent functions Q_V and Q_Z are the mean firing rates of the excitatory and inhibitory populations, respectively, also given by sigmoidal forms

$$Q_V(V) = 0.5Q_{V_{\max}}\left[1 + \tanh\left(\frac{V - V_T}{\delta_V}\right)\right], \tag{S5}$$

$$Q_Z(Z) = 0.5Q_{Z_{\max}}\left[1 + \tanh\left(\frac{Z - Z_T}{\delta_Z}\right)\right], \tag{S6}$$

where $Q_{V_{\max}}$ and $Q_{Z_{\max}}$ are the maximum firing rates of the excitatory and inhibitory populations, respectively, and V_T and Z_T are the corresponding thresholds for axon potential generation, and δ_V and δ_Z are the standard deviations in these thresholds.

Note that the inhibitory population Z does not possess conductance-based membrane dynamics, but rather is passively slaved to the pyramidal population: its membrane potential (and resulting firing rate) is driven by the firing rate of the output of the pyramidal cells, responding with the slow time scale parameterized by the factor b. The inhibitory population thus acts as a passive low-pass filter of the pyramidal cells. Even in the absence of a leaky current, the inhibitory neurons thus do not saturate,

but rather oscillate with the slow time scale of the system (approximately 1/10th of the fast pyramidal cells for the parameters used here). It is this mixing of time scales that leads to the chaos evident in Figures 1-3 of the main text.

Parameters are given in Table A1. To introduce additive noisy input to the excitatory population, we set $I_V = I_0 + \sigma\eta(t)$, where η is zero-mean Gaussian white noise and unit variance, and σ is the standard deviation of this component. To introduce multiplicative noisy input to the excitatory population, we set $I_V = I_0 + \sigma V(t)\eta(t)$.

Table S1: Parameters for neural mass model (in nondimensional units)

Parameter	Description	Value
g_{Ca}	Maximal conductance of calcium ion channel	1.1
g_{Na}	Maximal conductance of sodium ion channel	6.7
g_K	Maximal conductance of potassium ion channel	2
g_L	Conductance of passive leaky membrane	0.5
V_{Ca}	Equilibrium potential of calcium ion channel	1
V_{Na}	Equilibrium potential of sodium ion channel	0.53
V_K	Equilibrium potential of potassium ion channel	-0.7
V_L	Equilibrium potential of passive leaky membrane	-0.5
T_{Ca}	Threshold for calcium ion channels	-0.01
T_{Na}	Threshold for sodium ion channels	0.3

T_K	Threshold for potassium ion channels	0
δ_{Ca}	Threshold standard deviation for calcium ion channels	0.15
δ_{Na}	Threshold standard deviation for sodium ion channels	0.15
δ_K	Threshold standard deviation for potassium ion channels	0.3
V_T	Firing threshold potential for excitatory population	0
Z_T	Firing threshold potential for inhibitory population	0
δ_V	Firing threshold standard deviation for excitatory population	0.7
δ_Z	Firing threshold standard deviation for inhibitory population	0.7
$Q_{V_{\max}}$	Maximum firing rate for excitatory population	1
$Q_{Z_{\max}}$	Maximum firing rate for inhibitory population	1
b	Rate constant for inhibitory dynamics	0.1
ϕ	Rate constant for potassium dynamics	0.7
r_{NMDA}	Ratio of NMDA to AMPA receptors	0.25
a_{ee}	Synaptic strength from excitatory to excitatory	0.36
a_{ie}	Synaptic strength from inhibitory to excitatory	2
a_{ne}	Synaptic strength from nonspecific to excitatory	1
a_{ei}	Synaptic strength from excitatory to inhibitory	2
a_{ni}	Synaptic strength from nonspecific to inhibitory	0.4

I_0	Mean nonspecific input strength	0.3

Supplementary Information II: The Brain Dynamics Toolbox.

The Brain Dynamics Toolbox (https://bdtoolbox.blogspot.com.au/) is an open-source toolbox for simulating and exploring problems in dynamical systems using Matlab. It includes a graphical user interface with which users can explore the behaviour of a dynamical system in real-time by manipulating system parameters. It supports the most common classes of differential equations used in computational neuroscience; namely Ordinary Differential Equations, Delay Differential Equations and Stochastic Differential Equations.

The figures in the present paper were generated using version 2017c of the Brain Dynamics Toolbox. It can be downloaded from http://bdtoolbox.blogspot.com.

The time-series in Figure 1 were generated using the BTF2003 model which simulates a network of recurrently connected neural masses as described by Breakspear, Terry and Friston (Breakspear et al., 2003). The complete source-code for the model is shipped with the toolbox and can be run from the matlab command-line as follows:

```
>> cd bdtoolkit                     % bdtoolkit installation directory
>> addpath models                   % add the models to the matlab path
>> Kij = [0 1; 1 0];                % a 2x2 network connectivity matrix
```

```
>> sys = BTF2003(Kij);                    % construct the BTF2003 model
>> bdGUI(sys);                            % run the model in the GUI
```

The parameters and initial conditions of the model can be manipulated using the graphical controls on the right-hand side of the application window. For figure 1, all parameter values are the default with the exception of ($I = 0.2$; $\delta_z = 0.8, g_{Ca} = 0.9$) which put the system in a "faster" regime (and hence more clearly illustrate subsystem independence). These are reset by changing the corresponding parameter values and rerunning the simulation

```
>> sys.pardef(7).value(1,1)=0.9;          % calcium ion channel conductance gCa
>> sys.pardef(11).value=[0.8;0.8];        % std for inhibitory population δZ
>> sys.pardef(12)=0.2;                    % Mean nonspecific input strength I0
```

Panel A is replicated by the Time-Portrait panel of the graphical user interface. Panel C is replicated by the Hilbert Transform panel. Panel E is replicated by the Surrogate data panel available from the pull-down menus (New Panel - Surrogate Transform).

The time-series in Figure 2 were likewise generated using the BTF2003 model but in this case, the coupling has been "turned on" (C=0.1) and the default settings (Supplementary Information I, Table 1) are used for all parameters. The metric for

computing the non-linear prediction errors (panels F and G) are not included in the toolbox.

The simulations in Figures 4A-D were conducted using the FRRB2012 model which reproduces the canonical model of multistability proposed by (Frank Freyer et al., 2012). It too is shipped with the toolbox and can be run as follows:

```
>> n=1;                        % number of neurons to simulate
>> sys = FRRB2012(n);          % construct the FRRB2012 model
>> bdGUI(sys);                 % run the model in the GUI
```

The parameter values for Figure 4A were rho=0.65, eta=1, beta=-3 and lambda=4. Figure 4B used the same values except that beta=-1. Likewise for Figure 4C which had beta=-2. Figures 4E,G were simulated using the FRRB2012b model which is a network variant of the FRRB2012 model that is also shipped with the toolbox. It can be replicated as follows:

```
>> Kij = [-1 1; 1 -1];         % network connectivity matrix
>> sys = FRRB2012b(Kij);       % construct the FRRB2012b model
>> bdGUI(sys);                 % run the model in the GUI
```

Where the global coupling coefficient was c=0 and c=0.5 in Figures 4E and 4G respectively. All other parameters were lambda=4, beta=-2, eta=1, rho=0.65.

The simulations in Figure 5 were conducted using the BTF2003DDE model which is a time-delayed variant of the BTF2003 model (Breakspear et al., 2003). In this case it uses the 47x47 macaque connectivity matrix from the Brain Connectivity Toolbox which can be loaded as follows:

```
>> load macaque47.mat CIJ          % CIJ is a 47x47 connectivity matrix
>> sys = BTF2003DDE(CIJ);          % construct the BTF2003DDE model
>> bdGUI(sys);                     % run the model in the GUI
```

The coupling strength and time delay can be manipulated with the options in GUI.

Supplementary Information III: Bistable Hopf model

Full details of this model can be found in (Frank Freyer et al., 2012). The version here is adapted from (Roberts et al., 2017).

This model is a type of *normal form* model that describes a generic limit cycle oscillator with bistability (Frank Freyer et al., 2012). Normal forms describe the dynamics near a bifurcation, and are useful because all instances of that bifurcation (whatever the system) can be reduced to essentially the same simple canonical form. Depending on the choices of its parameters, the bistable Hopf model describes a fixed point (stable or unstable) and 0, 1, or 2 limit cycles. A Hopf bifurcation is a type of bifurcation where a fixed point loses stability, giving rise to a limit cycle (also termed a periodic orbit). A model with a limit cycle is often termed an "oscillator". There are two types of Hopf bifurcation: supercritical, where the limit cycle is stable and coexists with the unstable fixed point, and subcritical, where the limit cycle is unstable and coexists with the stable fixed point. The Hopf normal form is most conveniently expressed in polar coordinates (r, θ), where r is the amplitude (so $r = 0$ corresponds to a fixed point), and θ is the phase. To drive a transition from the fixed point to the limit cycle (i.e., "seizure onset"), we include independent additive $b_1.\zeta_1$ and multiplicative $b_2.\zeta_2 x$ noisy drives, where ζ_1 and ζ_2 are zero-mean unit-variance Gaussian white noise, and $b_1$and b_2 are the standard deviations of the stochastic inputs.

The state variables evolve according to,

$$\frac{dr}{dt} = -r^5 + \lambda r^3 + \beta r + \sigma\eta(\square), \tag{S7}$$

$$\frac{d\theta}{dt} = \omega, \tag{S8}$$

where λ and β are parameters that determine the number, amplitude, and stability of limit cycle solutions, ω is a constant angular velocity (in this simple case, the phase plays no role in the dynamics – it is directly proportional to time). For the bistable model here, we have extended the more typical 3rd-order Hopf bifurcation normal form to 5th order. This has the effect of introducing an additional high-amplitude limit cycle that coexists with the low amplitude one, as well as an additional bifurcation (a "saddle-node") where two solutions meet and annihilate. This form is bistability generic, in the sense that it emerges from a normal form that exists near any Hopf bifurcation.
